# Survival Time after Surgical Debulking and Temozolomide Adjuvant Chemotherapy in Canine Intracranial Gliomas

**DOI:** 10.3390/vetsci9080427

**Published:** 2022-08-12

**Authors:** Emma Hidalgo Crespo, Alba Farré Mariné, Martí Pumarola i Battle, Juan Francisco Borrego Massó, Alejandro Luján Feliu-Pascual

**Affiliations:** 1 AÚNA Especialidades Veterinarias, Calle Algepser 22-1, 46980 Paterna, Valencia, Spain; 2 Department of Animal Medicine and Surgery, Veterinary Faculty, Universitat Autonoma de Barcelona, 08193 Bellaterra (Cerdanyola del Vallès), Barcelona, Spain

**Keywords:** chemotherapy, glioma, intracranial tumour, surgery, survival time, treatment

## Abstract

**Simple Summary:**

Infiltrative brain tumours are common in dogs. Although different treatments have been used, such as surgery, radiotherapy, chemotherapy, or combinations, guidelines for the most effective management are lacking. In this study, we report the effect of combining surgery and chemotherapy on the survival of 14 dogs with infiltrative gliomas. Four dogs were operated on two or three times to remove the tumors, and only one of these dogs died shortly after the second surgery. All tolerated the surgery with minimal or no deterioration, and all were euthanized between 6 months to 2 years after diagnosis due to tumour progression. To conclude, surgery and chemotherapy, although not curative, can prolong survival in dogs with infiltrative brain tumours. This information may help future research into the most appropriate treatment for this debilitating condition.

**Abstract:**

Intracranial gliomas are associated with a poor prognosis, and the most appropriate treatment is yet to be defined. The objectives of this retrospective study are to report the time to progression and survival times of a group of dogs with histologically confirmed intracranial gliomas treated with surgical debulking and adjuvant temozolomide chemotherapy. All cases treated in a single referral veterinary hospital from 2014 to 2021 were reviewed. Inclusion criteria comprised a histopathological diagnosis of intracranial glioma, adjunctive chemotherapy, and follow-up until death. Cases were excluded if the owner declined chemotherapy or there was insufficient follow-up information in the clinical records. Fourteen client-owned dogs were included with a median time to progression (MTP) of 156 days (95% CI 133–320 days) and median survival time (MST) of 240 days (95% CI 149–465 days). Temozolomide was the first-line adjuvant chemotherapy but changed to another chemotherapy agent (lomustine, toceranib phosphate, or melphalan) when tumour relapse was either suspected by clinical signs or confirmed by advanced imaging. Of the fourteen dogs, three underwent two surgical resections and one, three surgeries. Survival times (ST) were 241, 428, and 468 days for three dogs treated twice surgically and 780 days for the dog treated surgically three times. Survival times for dogs operated once was 181 days. One case was euthanized after developing aspiration pneumonia, and all other cases after progression of clinical signs due to suspected or confirmed tumour relapse. In conclusion, the results of this study suggest that debulking surgery and adjuvant chemotherapy are well-tolerated options in dogs with intracranial gliomas in which surgery is a possibility and should be considered a potential treatment option. Repeated surgery may be considered for selected cases.

## 1. Introduction

Intracranial neoplasms are common in dogs [1] and represent the major neurological cause of mortality and morbidity in companion animals [2,3]. The incidence of intracranial neoplasia in dogs based on necropsy data has been reported to be 2–4.5% [1,4], while the prevalence of primary intracranial tumours in dogs is 2.3% [1]. Breeds like Boxer, Boston Terrier, Golden retriever, French bulldog, and Rat terrier showed a higher incidence of primary intracranial neoplasms [1]. In contrast, Cocker spaniel or Doberman pinscher showed a significantly lower risk of primary intracranial neoplasms [1]. Astrocytomas and oligodendrogliomas are highly overrepresented in specific brachycephalic breeds (Boxers, Boston terriers, and Bulldogs) [1,3,4,5,6,7,8], with over 50% of all canine gliomas occurring in dogs with this head conformation [1,2,5,6].

The distribution of intracranial neoplasia in dogs is divided into 50% primary tumours and 50% secondary tumours [1]. Meningiomas represent around 50% of primary intracranial neoplasia in dogs, gliomas 36%, and choroid plexus tumours are the third most common [1,3,4,5,9,10]. Secondary intracranial tumours, in order of frequency, are hemangiosarcoma, pituitary tumours, lymphoma, metastatic carcinoma, an extension of nasal neoplasms, and histiocytic sarcoma [11].

Intracranial neoplasms are more frequently seen in adult dogs over 5 years of age [1,8,10,11,12], with gliomas diagnosed at a median age of 8 years [1,2,3,4,5,10], which are predominantly located in the olfactory bulb, temporal and parietal lobes [1,2,5].

A presumptive diagnosis of an intracranial tumour is established based on history, clinical signs, and advanced imaging [2,9,13,14,15,16,17]. However, the definitive diagnosis must be based on histopathological examination [3,13,14] of tissue samples according to the World Health Organization (WHO) classification [12,18]. One study has demonstrated a low accuracy of 53.3% and 60% for predicted tumour grade and type, respectively, based on MRI features and histopathology [19]. Moreover, high-field MRI has demonstrated sensitivities of 0–91.1% and specificities ranges of 84.8–99.3% for histopathologically confirmed intracranial lesions [20]. Therefore, caution should be paramount when comparing diagnostic features and outcomes, particularly between studies with presumptive and confirmed intracranial tumours.

Several studies have reported survival times of presumptive or confirmed gliomas following palliative treatment, surgical debulking, chemotherapy, and radiotherapy, either alone or in combination [3,21,22]. Regardless of the treatment used, intracranial gliomas are associated with a poor prognosis, and the most effective treatment is yet to be defined [3,21,22]. Due to this circumstance, and the lack of histopathological type and grade of the tumour, it is difficult for the owner to opt for a specific treatment, a combination, or euthanasia.

There are, however, no reported cases of gliomas treated with surgery followed by a combination of different oral chemotherapies (temozolomide alone or temozolomide combined with other chemotherapy agents) or surgical reintervention.

The primary objective of this descriptive retrospective study is to report the times of progression and survival times of a group of dogs with histologically confirmed intracranial gliomas treated with debulking surgery and adjuvant temozolomide chemotherapy. The secondary objective is to describe the effects of surgical reintervention in the survival time as a treatment option after tumour relapse.

## 2. Materials and Methods

### 2.1. Case Selection and Data Collection

Cases with histopathologically diagnosed intracranial gliomas treated with surgical debulking followed by temozolomide chemotherapy were identified by searching the medical record database of a single veterinary referral hospital from 2014 to 2021. Informed written consent was obtained from the owners for diagnostic procedures and surgical interventions. Inclusion criteria were confirmed histopathological diagnosis, tumour debulking surgery, adjunctive chemotherapy treatment, and follow-up data until death. Cases were excluded from the retrospective analysis if the owner had declined chemotherapy, and if there was inadequate information available to the primary investigators, specifically including a lack of detail regarding the diagnostic, staging, and monitoring tests.

### 2.2. Medical Records

Data obtained from the medical records included the dog’s age, gender, reproductive status, breed, neurological signs, magnetic resonance imaging (MRI) when available and computed tomography (CT) findings pre-and post-surgery, tumour location, surgical approach, temozolomide chemotherapy dose received, histologic tumour type and grade, time to progression, additional surgeries or chemotherapy rescue protocols, date and cause of death. The referring veterinarian(s) and/or owner(s) were contacted to follow up where additional detail was required.

A presumptive diagnosis of glioma was based on the neurological localization, pre-surgical CT with iodinated contrast 400 mg/kg IV (Omnipaque 300, General Electric’s Healthcare, Madrid, Spain), or MRI images with gadolinium contrast IV and cerebrospinal fluid (CSF) analysis. Neurological examination and surgical debulking were performed by the same board-certified veterinary neurologist. During the surgical procedure, biopsy samples were fixed in 10% formalin and submitted for histopathological analysis to confirm the diagnosis. An immediate CT scan of the brain (Brivo CT385 General Electric’s Healthcare, Madrid, Spain) was performed to assess the extent of macroscopic resection. Biopsy samples were reviewed by a board-certified veterinary pathologist and classified according to the WHO criteria [18].

After histopathological confirmation, adjuvant temozolomide (Temodal^®^, Merck Sharp and Dohme, Haarlem, The Netherlands) chemotherapy (76.4–181.8 mg/m^2^/24 h for 5 days every 3 weeks) was prescribed in all dogs [23,24]. Toxicities were graded according to the Veterinary Cooperative Oncology Group’s Common Terminology Criteria for Adverse Events (VCOG-CTCAE) following investigational therapy in dogs and cats v1.2 [25].

### 2.3. Statistical Analysis

Kaplan-Meier (KM) product limit estimates [26] and 95% CI were calculated for time to progression and survival times. The time to progression was calculated from the day of imaging diagnosis until the progression of neurological clinical signs compatible with tumour regrowth or advanced imaging confirmation. Death was attributed to recurrence of the tumour if there was imaging confirmation or neurological deterioration, and the neurolocalization was consistent with the previous tumour location. Censoring was applied in cases where death was not related to the tumour. If no information on the cause of death was available or the case was lost in follow-up, it was assumed to be related to the tumour. Statistical analysis was performed using Prism 9 (GraphPad Software, San Diego, CA, USA).

## 3. Results

Seventy-six dogs with presumptive gliomas based on advanced imaging characteristics (CT or MRI) were diagnosed during the study period, (2014–2021) but lacked histopathological confirmation. Fifteen additional dogs with histopathological diagnosis of intracranial glioma were intervened during the same period. One dog was censored from survival time analysis because it was euthanatized due to aspiration pneumonia in the postoperative period after the second surgery, and another dog was excluded because the owner had declined chemotherapy. A total of 14 dogs were therefore included in this study (Table 1). The most represented breed was the French bulldog (*n* = 10/14), with the Boxer (*n* = 2/14), English bulldog (*n* = 1/14), and Boxer mix (*n* = 1/14) marginally represented, all of them brachycephalic in conformation. There were 9/14 females (8/9 neutered and 1/9 entire) and 5/14 males (4/5 neutered and 1/5 entire). The median age at the time of diagnosis was 8 years (range: 4–10 years). The median weight was 14 kg (range 8.9–37.2 kg).

Neurological examination at the time of initial presentation was available in all dogs. The most common neurological signs in order of frequency included seizures (n = 13/14 dogs, 92.9%), followed by obtundation (n = 3/14, 21.4%), circling (n = 3/14, 21.4%), proprioceptive deficits (n = 7/14, 50%) and cranial nerve deficits (n = 6/14, 42.9%) including decreased menace response (n = 5/14, 35.7%). In three dogs, neurological examination at the time of first evaluation was considered normal.

**Figure 1:**
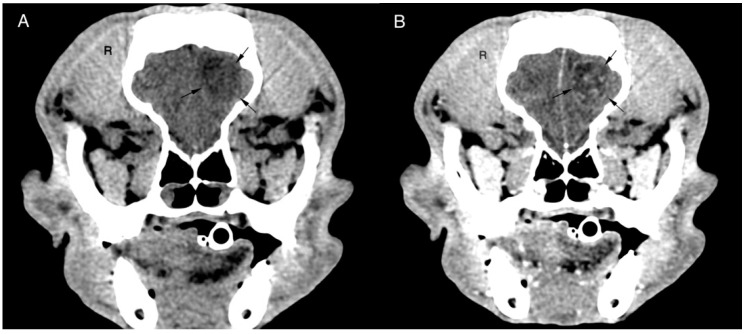
Transverse pre (**A**) and post (**B**) contrast CT brain image with soft tissue algorithm. The images reveal an intra-axial, ill-defined round, hypoattenuating lesion located in the left frontal lobe of the cerebrum. The lesion shows ring enhancement after ionated contrast administration and a slight midline shift to the right. Histopathological diagnosis was oligodendroglioma grade II.

In 13/14 dogs, the tumour had a rostrotentorial location (92.9%) and caudotentorial location in 1/14 dogs (7.1%). For rostrotentorial tumours, the frontal lobe (n = 7/14, 50%) was most affected with other localizations such as temporal lobe (n = 3/14, 21.4 %), olfactory bulb (n = 1/14, 7.1%), parietal lobe (n = 1/14, 7.1%) and occipital lobe (n = 1/14, 7.1%) less commonly observed. The only caudotentorial tumour was in the cerebellum (n = 1/14, 7.1 %) (Figure 1).

All dogs (14/14) were treated perioperatively with prednisone 0.5 mg/kg twice daily (Prednisone Alonga^®^, Famar Health Care, Madrid, Spain) and, when seizures were present (n = 13/14), with additional antiepileptics drugs such as phenobarbital 3–6 mg/kg twice daily (Luminal^®^, Kern Pharma, Barcelona, Spain) or/and levetiracetam 20–40 mg/kg three times a day (Keppra^®^, UCB Pharma SA, Brussels, Belgium). Maropitant 1 mg/kg IV (Cerenia^®^ 10 mg/mL Zoetis, Madrid, Spain) was used preoperatively to prevent perianaesthetic vomiting. During the surgery, all dogs received mannitol 0.5 g/kg IV (Osmofundina concentrada 20%^®^, BBraun Medical S.A, Rubí, Spain); nine dogs received cefazoline 20 mg/kg IV three times a day (Cefazolina 1 g Normon^®^, Madrid, Spain) and five dogs received trimethoprim-sulfamethoxazole 15 mg/kg IV twice daily (Soltrim^®^, Almirall S.A., Barcelona, Spain) when the surgical approach was through the frontal sinus.

Initial surgical approaches were selected according to the location of the tumour and included transfrontal (n = 9/14, 64.3%), temporal (n = 3/14, 21.4%), parietooccipital (n = 1/14, 7.1%) and suboccipital (n = 1/14, 7.1%) areas. In all dogs, surgical debulking was aimed at a total macroscopic resection. Bone flap or titanium mesh to replace the excised bone was not used in any dog. Four dogs were reintervened when tumour relapse was suspected based on advanced imaging findings. One dog was operated on three times: twice with a transfrontal approach and once frontoparietal when the tumour extended caudally. Three dogs were surgically treated twice: two transfrontally, and in the third case, an initial suboccipital approach was combined with a parietooccipital approach during the second surgery.

Following surgical discharge, all dogs were treated with a dose of prednisone 0.5 mg/kg twice daily, which was gradually decreased. Dogs with seizures (13/14) also received antiepileptic drugs, phenobarbital 3–6 mg/kg twice daily or/and levetiracetam 20–40 mg/kg three times a day.

Short-term surgical complications included subclinical pneumocephalus after the surgery in all patients, and aspiration pneumonia two days after the surgery leading to euthanasia in one patient. Neurological status following surgery was unchanged or showed mild deterioration, but all dogs were ambulatory within 24 h post-surgery. Other life-threatening complications or long-term neurological deterioration were not encountered.

Using the WHO Grading classification of Tumours of the Central Nervous System [18], the neoplasia type resected during the first surgery included oligodendroglial tumours (n = 12/14, 85.7%) and astrocytic tumours (2/14, 14.3%). The oligodendroglial lineage included oligodendroglioma grade II (n = 4/12, 33.3%) and anaplastic oligodendroglioma grade III (n = 8/12, 66.7%). Both astrocytic tumours were glioblastoma grade IV (n = 2/2, 100%).

Four dogs were reintervened surgically (4/14): once in three dogs (3/4) and twice in one dog (1/4). In three reintervened dogs, the tumour type did not change (oligodendroglioma grade III in one dog was operated on twice and in the one operated on three times, and oligodendroglioma grade II in the other one operated on twice). Only in one dog operated on twice did the tumour type change from anaplastic oligodendroglioma grade III after the first surgery to gliosarcoma grade IV after the second surgery.

After histopathological confirmation, adjuvant chemotherapy was prescribed. All dogs received temozolomide (Temodal^®^, Merck Sharp and Dohme, Haarlem, The Netherlands) 76,4–181,8 mg/m2/24h for 5 days every 3 weeks within 1–3 weeks after the first surgery. Nine dogs (9/14) received only temozolomide, with the remaining five dogs (5/14) receiving additional rescue chemotherapy after a confirmed or suspected tumour relapse.

In two dogs receiving rescue chemotherapy (2/5), the treatment was changed to lomustine (CeeNU^®^, Bristol-Myers Squibb, Princeton, NJ, USA) at 70 mg/m^2^ every 3 weeks (one suspected relapse after CT imaging and one confirmed after a second tumour debulking and histopathology). Whereas in two dogs (2/5), chemotherapy was changed to toceranib phosphate (Palladia^®^, Zoetis, Kalamazoo, MI, USA) 2.5–2.8 mg/kg 3 times weekly (one suspected relapse after CT imaging and the other confirmed after a second tumour debulking and histopathology). In this last dog reintervened surgically, a new relapse was suspected while it was receiving toceranib phosphate and underwent a third surgery. At that stage, toceranib phosphate was replaced by lomustine 70 mg/m^2^ every 3 weeks. Finally, in the last dog (1/5), temozolomide was replaced with melphalan (Alkeran^®^, Aspen Pharmacare S.L., Dublin, Ireland) at 0.15 mg/kg for 5 days every 3 weeks after the dog showed signs of clinical deterioration without tumour relapse confirmation. In the dog euthanatized two days after the second surgery due to aspiration pneumonia, two postoperative CT scans within 48 h failed to show an intracranial haemorrhage or brain oedema as the cause of deterioration.

Side effects of chemotherapy protocols were only observed in one dog with petechiae, haematuria, and pancytopenia (grade 2) [25] after the second temozolomide cycle. Clinical and laboratory abnormalities were resolved six weeks after temozolomide discontinuation, and tumour recurrence was observed after a control CT at that time.

**Figure 2:**
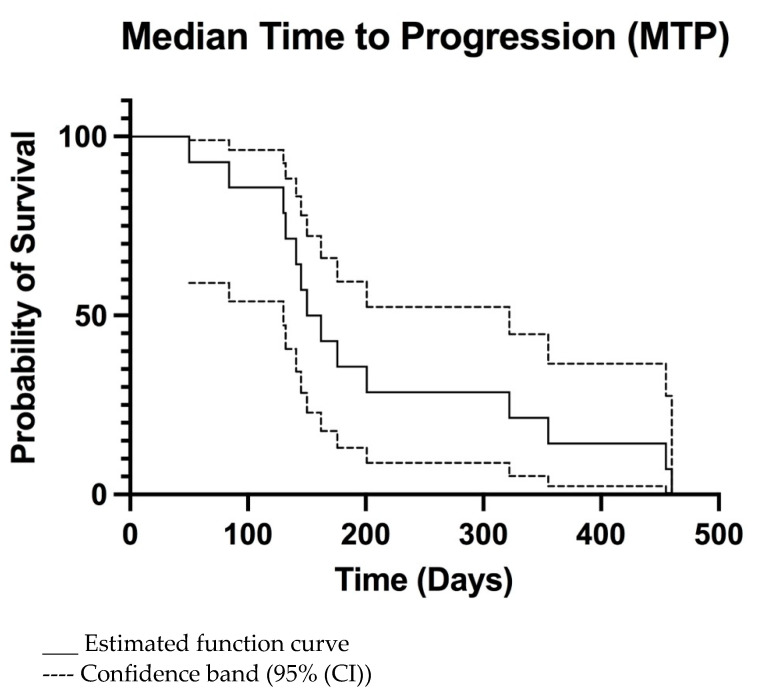
Kaplan-Meier median time to progression (MTP) curve of all dogs.

**Figure 3:**
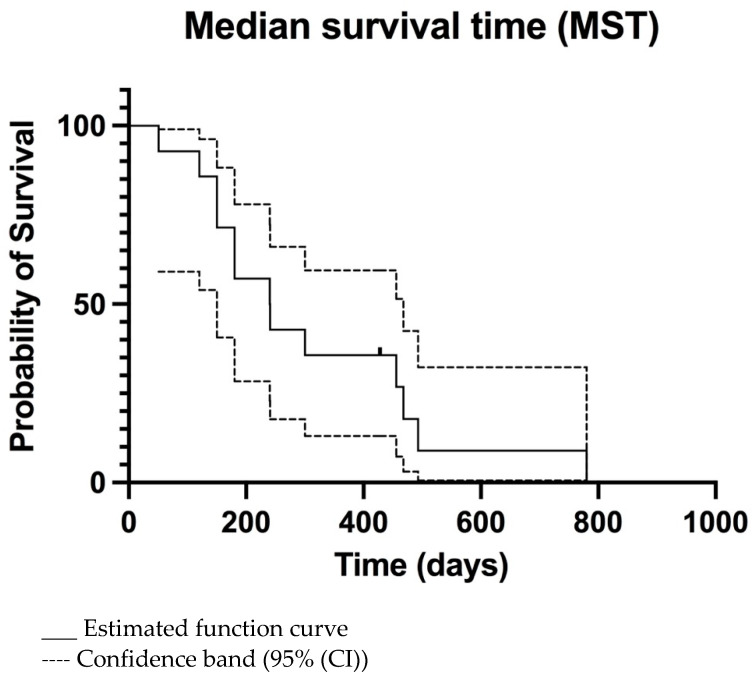
Kaplan-Meier median survival time (MST) curve of all dogs. The only censored patient is marked.

The overall MTP was 156 days (95% CI 133–320 days) (Figure 2) and MST was 240 days (95% CI 149–465 days) (Figure 3). For dogs treated surgically twice, ST was 241, 428, and 468 days. For the dog treated surgically three times, ST was 780 days. The dog euthanatized two days after the second surgery due to aspiration pneumonia was censored from the survival analysis. Dogs surviving the immediate postoperative period (13/14) were euthanatized, 3/13 due to histopathologically confirmed tumour relapse, 2/13 after observed tumour recurrence in CT imaging, and 8/13 after neurological deterioration for suspected tumour relapse.

## 4. Discussion

Common therapeutic options available for veterinary patients with intracranial neoplasia include palliative care, surgery, chemotherapy, and radiotherapy, either alone or combined [3,21,22].

For histologically confirmed gliomas, therapies and outcomes vary between studies. Those describing palliative treatment (glucocorticoids, antiepileptic drugs, and analgesics) included 22 dogs, where the survival time ranged between 1–492 days [2,27]. In one recent study, including 14 dogs treated with surgery alone, the MST was 66 days (range: 10–730 days) [9]. With a combination of surgery and radiotherapy, the MST increased to 300 days (range: 60–405 days) according to another study with 13 dogs [27]. The use of surgery and intratumoral chemotherapy (chlorambucil and lomustine) in 8 dogs achieved an MST of 257 days [28]. In one case report of an anaplastic oligodendroglioma treated with radiotherapy and lomustine, the dog lived for 910 days [29], whereas intratumoral temozolomide was used in three dogs, but long-term survival time was not reported [30].

In cases of presumptive diagnoses, treatment with radiotherapy achieved MST of 255 and 636 days in a total of 33 dogs in two different studies [16,27], while in another two studies, including 96 dogs treated with oral lomustine, MST of 93 and 138 days were reported [31,32] (Table 2).

However, there is a paucity of clinically relevant data on type-specific therapeutic outcomes for canine intracranial neoplasia [2,3,21]. In addition, current experimental therapeutic studies lack comparison between different tumour types or grades [14,28,33,34].

Previous studies have observed a breed association of specific neoplasms such as gliomas with brachycephalic breeds [1,4]. This finding is in accordance with our results, where all the dogs had this head conformation. Future genetic investigations might shed some light on the underlying causes of this association.

Most published clinical studies evaluating outcomes for intracranial gliomas in veterinary patients are based on diagnostic imaging characteristics and lack histopathological confirmation [16,17]. Additional single cases or case series also have this important limitation [31,32,35]. Considering that the accuracy of MR imaging for predicting tumour type has been reported as 70% [36], some of the cases included in those studies might not represent true gliomas, and the survival time might be erroneously prolonged in non-neoplastic lesions. In one recent article, the MRI accuracy to distinguish between gliomas and meningoencephalitis of unknown origin (MUO) was found to have a sensitivity between 76–89% and a specificity between 77–81% after processing texture analysis [37]. Another study demonstrated 94.4% sensitivity and 95.5% specificity of MRI detecting a brain lesion for classifying neoplastic and inflammatory disease [20]. To allow a more accurate comparison with our results, our discussion will centre on surgically confirmed or post-mortem cases from the previous veterinary literature.

In human medicine, first-line management of intracranial gliomas includes maximal surgical resection followed by radiotherapy and six cycles of maintenance temozolomide chemotherapy [38,39]. Lomustine probably remains the most commonly drug used after temozolomide for glioma treatment. However, lomustine is regarded as the main standard of care for recurrent glioblastoma in Europe [38].

Currently, the preferred treatment option for gliomas in veterinary medicine is radiotherapy; however, most published studies using this treatment modality lack histopathological confirmation [16,17,35]. Due to the limited availability of radiotherapy equipment, the need for repeated anaesthesia, and side effects, other therapeutic options have also been explored [3,9,17,21,28,31,32].

Clinical data of intracranial gliomas treated with chemotherapy is limited [2,3,21,28,29,30,31,32], but overall, the outcome results are disappointing [2,3,17,28,29,30,31,32]. As mentioned previously, the main drawback when comparing the scarcity of published clinical studies is the lack of histopathological analysis of biopsy samples when chemotherapy has been used [31,32]. In addition, these studies usually involve a small number of cases, and the protocols, including chemotherapy agents and doses, are variable.

The most frequently used chemotherapeutic drugs for confirmed gliomas are alkylating agents such as lomustine, carmustine, temozolomide, or antimetabolite hydroxyurea, all of which penetrate the blood-brain barrier [2,3,28,29,30]. Studies using either of these agents in confirmed cases have reported an MST of 56 days only with chemotherapy treatment [2] or 206 and 257 days using chemotherapy and surgical treatment in two different studies [2,28]. In veterinary medicine, the reported dose used for temozolomide is 60–100mg/m^2^ for 5 days every 3 weeks [23,24]. These survival times are comparable with dogs in our study who underwent one surgical debulking and temozolomide chemotherapy, but is shorter than in dogs who underwent two or three surgeries and a chemotherapy protocol change.

Surgical resection only with curative intent has been used to treat intracranial gliomas in two studies with an MST of 66 [9] and 114 days [2]. For our subset of dogs operated on once followed by temozolomide, the MST was 181 days (95% CI 53–444 days), which compares favourably when only chemotherapy [2] or surgery was used [2,9].

Reoperating for recurrent low-grade gliomas has improved the MST in human medicine [40]. Moreover, for aggressive glioblastomas, surgical reintervention is used to prolong the survival time in selected human patients once radiotherapy or chemotherapy fails to control its growth. To the authors’ knowledge, surgical reintervention has never been reported in canine gliomas, and only two dogs with intracranial meningiomas have been reoperated [9]. In our case series, survival times were 241, 428, and 468 days for dogs treated twice surgically, whereas the dog treated surgically three times survived for 780 days after diagnosis. These results show a longer survival time than dogs with a single surgical debulking. It is possible that both repeating surgical debulking and changing the chemotherapy agent contributed to achieving these results. Another possible explanation is that committed owners are open to trying another surgery and protocol given the good recovery from the first debulking and lack of side effects from the chemotherapy in their dogs. Although the number of reoperated dogs is low, our results open the possibility of exploring this option for selected cases.

For radiotherapy in gliomas confirmed either surgically or post-mortem, previous studies have reported MST of 94 [2] and 300 days [27]. Anecdotally, a single canine case with a post-mortem diagnosis of anaplastic oligodendroglioma was treated with radiotherapy and lomustine and lived for 910 days [29]. Repeated radiotherapy is increasingly used in human patients with recurrent gliomas; however, this approach poses the risk of radiation necrosis if the tolerance of normal brain tissue has exceeded, leading to neurological signs similar to those caused by tumour recurrence or the development of radiation-induced tumours [41]. In one study, radiation necrosis was suspected as the cause of death or euthanasia in 14.5% of the dogs included (12/83) [41].

The cause of death in our study was tumour-related euthanasia due to histopathologically confirmed tumour relapse (3/13), after an observed tumour recurrence in CT imaging (2/13), and after neurological deterioration for suspected tumour relapse (8/13). Only one dog was euthanatized in the immediate postoperative period due to aspiration pneumonia after the second surgery. This dog recovered from the anaesthetic procedure but developed a laboured respiratory pattern and respiratory acidosis within five hours, requiring intermittent ventilation. In addition to the immediate postoperative CT scan, two additional CT scans within 48 h failed to show intracranial haemorrhage or brain oedema as the reason for the deterioration; therefore, it was attributed to its brachycephalic conformation. Aspiration pneumonia has been reported as the most common non-neurological postoperative complication (12%) after intracranial surgery, but these studies have not shown a higher incidence in brachycephalic breeds [42,43,44].

Temozolomide for the doses administered in our study appears safe to use in dogs with confirmed intracranial gliomas. In the only dog with associated side effects, those ceased after discontinuation and supportive care. It remains unclear whether higher temozolomide doses might have been more effective in controlling tumour regrowth without increasing the probability of side effects. As a result, the combination treatment of our study (surgery and chemotherapy) appears to be a safe therapeutic option with longer MST than those reported for palliative treatment of canine brain tumours with an ST range of 7–70 days [21,45] or surgical resection only with MST of 66 days [9] in one study and 114 days [2] in another study. Moreover, in dogs operated twice or three times, the MST increased compared to the time of imaging recurrence, which makes repeated resections of recurrent gliomas an approach to consider when enough viable brain tissue remains to ensure a good quality of life.

The main limitations of the study include the limited number of cases, its retrospective nature, and the combination of surgical debulking and adjuvant chemotherapy without standardised protocols. Direct comparison of survival times for such heterogeneous treatments becomes complicated, and it is difficult to determine the exact influence of each modality used in the outcome. In addition, variables such as age, severity, and duration of neurological signs, location of the neoplasm (rostrotentorial vs. caudotentorial), and adjuvant chemotherapy protocol administered (single or multiple chemotherapies) might have influenced the MST. In this study, the low number of cases precludes any strong conclusion but opens the door to exploring a multitherapeutic approach in future clinical research.

**Table 2:** Median survival times of canine glioma treatment studies to date with treatment modalities and comparison with the results of our study.

Study	Animals Number	Tumour	Diagnosis	Chemotherapy/Radiotherapy Treatment	Surgery	Median ST (Days)
			**Presumptive**			
**Van Meervenne, S. et al. (2012)** [31]	56 dogs	Intra or extra-axial	Presumptive (CT)	Lomustine	No	93
**Moirano, S. et al. (2018)32**	40 dogs	Gliomas	Presumptive (MRI)	Lomustine	No	138
**Magalhães, TR. et al. (2021)** [35]	16 dogs	Gliomas	Presumptive (MRI)	Radiotherapy	No	512
**Dolera, M. et al. (2017)** [17]	22 dogs	Gliomas	Presumptive (MRI)	Radiotherapy	No	383
**Dolera, M. et al. (2017)** [17]	20 dogs	Gliomas	Presumptive (MRI)	Radiotherapy and temozolomide	No	420
**Moirano, S. et al. (2020)** [16]	5 dogs	Gliomas	Presumptive (MRI)	Radiotherapy and chemotherapy (lomustine, hydroxyurea, temozolomide or toceranib phosphate)	No	636
			**Necropsy**			
**José-López, R. et al. (2021)** [2]	12 dogs	Gliomas	Definitive (MRI and necropsy)	Chemotherapy (lomustine, temozolomide, cytarabine and clinical trial drug)	No	56
**Adams et al. (2005)** [27]	12 dogs	Gliomas	Definitive (MRI and necropsy)	Radiotherapy	No	255
**José-López, R. et al. (2021)** [2]	1 dog	Glioma undefined (high-grade)	Definitive (MRI and necropsy)	Radiotherapy	No	29
**Hasegawa, D. et al. (2012)** [29]	1 dog	Anaplastic Oligodendroglioma	Definitive (MRI and necropsy)	Radiotherapy and lomustine	No	910
			**Biopsy**			
**Suñol, A. et al. (2017)** [9]	14 dogs	Gliomas (8 oligodendrogliomas, 4 astrocytomas, and 2 anaplastic gliomas)	Definitive (MRI and biopsy)	No	Yes	66
**José-López, R. et al. (2021)** [2]	4 dogs	Oligodendroglioma	Definitive (MRI and biopsy)	No	Yes	125 days
**José-López, R. et al. (2021)** [2]	3 dogs	Oligodendroglioma (high-grade)	Definitive (MRI and biopsy)	Chemotherapy (lomustine and temozolomide)	Yes	206 days
**Hicks, J. et al. (2019)** [30]	4 dogs	Gliomas (1 gliomasglioblastoma multiforme and 3 oligodendroglioma)	Definitive (MRI and biopsy)	Intratumoral temozolomide microcylinders	Yes	Not reported
**Bentley, R. et al. (2018)** [28]	8 dogs	Gliomas (7 oligodendrogliomas and 1 astrocytoma (6 high grade, 2 low grade)	Definitive (MRI and biopsy)	Chlorambucil and lomustine	Yes	257 days
**Adams et al. (2005)** [27]	13 dogs	Gliomas	Definitive (MRI and biopsy)	Radiotherapy	Yes	300
**José-López, R. et al. (2021)** [2]	1 dog	Glioma undefined (high-grade)	Definitive (MRI and biopsy)	Radiotherapy	Yes	94 days
**Our study**	14 dogs	Gliomas (4 oligodendroglioma, 8 anaplastic oligodendroglioma and 2 glioblastoma grade IV)	Definitive (MRI or CT and biopsy)	Temozolomide and toceranib phosphate, lomustine and/or melphalan	Yes	240 days

ST = survival time, MRI = magnetic resonance imaging, CT = computed tomography.

## 5. Conclusions

Despite the limitations of the study, including the low number of cases and its retrospective nature, combined surgical debulking and adjuvant chemotherapy in canine intracranial gliomas is a feasible therapeutic option, in particular for countries where radiation facilities are lacking or their availability is scarce. We observed a longer MST compared to previous literature using different approaches, such as surgery or chemotherapy alone, which might settle the base for additional research to set up protocols for effective treatments. In addition, surgical reintervention increased survival time in our cases, which should be investigated in future studies.

## Figures and Tables

**Table 1:** Clinical data of dogs diagnosed with gliomas treated with surgery and chemotherapy.

Breed	Sex	Neutered (Y/N)	Age (yrs)	Weight (kg)	Primary Complaint	Neurological Exam	Tumour Localization	Surgery (N)	Histopathology	Chemotherapy	Tumour Relapse	Survival Time (Days)	Cause Death
**French Bulldog**	M	Y	8	13.0	Seizures	Left circling and right proprioceptive deficits	Left temporal lobe	1	Glioblastoma grade IV	Temozolomide (181.8 mg/m^2^ for 5 days every 3 weeks)	Suspected	456	Euthanasia due to clinical signs of relapse
**French Bulldog**	F	Y	8	9.8	Seizures	Obtunded mental status, right circling, ambulatory tetraparesis, decreased left menace response and facial sensation	Right frontal lobe	1	Anaplastic oligodendroglioma grade III	Temozolomide (109.0 mg/m^2^ for 5 days every 3 weeks)	Suspected	190	Euthanasia due to clinical signs of relapse
**French Bulldog**	M	N	9	14.6	Seizures	Normal	Right temporal lobe	1	Oligodendroglioma grade II	Temozolomide (100.0 mg/m^2^ for 5 days every 3 weeks)	Suspected	300	Euthanasia due to clinical signs of relapse
**French Bulldog**	F	Y	9	9.2	Seizures	Decreased menace response OS	Right temporal lobe	1	Glioblastoma grade IV	Temozolomide (93.0 mg/m^2^ for 5 days every 3 weeks)	Suspected	150	Euthanasia due to clinical signs of relapse
**Boxer**	F	Y	10	37.2	Seizures	Obtunded mental status, right circling, ambulatory tetraparesis, decreased left menace response and facial sensation	Left frontal lobe	1	Anaplastic oligodendroglioma grade III	**1st** Temozolomide (110. mg/m^2^ for 5 days every 3 weeks) **2nd** Melphalan (0.15 mg/kg for 5 days every 3 weeks)	Suspected	493	Euthanasia due to clinical signs of relapse
**French Bulldog**	F	Y	10	15.3	Seizures	Mild right proprioceptive deficits	Left frontal lobe	1	Oligodendroglioma grade II	Temozolomide (100.0 mg/m^2^ for 5 days every 3 weeks)	Suspected	51	Euthanasia due to clinical signs of relapse
**French Bulldog**	M	N	7	14.0	Seizures	Normal	Left frontal lobe	1	Anaplastic oligodendroglioma grade III	Temozolomide (76.9 mg/m^2^ for 5 days every 3 weeks)	Suspected	160	Euthanasia due to clinical signs of relapse
**French Bulldog**	M	N	7	13.5	Seizures	Normal	Left frontal lobe	1	Anaplastic oligodendroglioma grade III	Temozolomide (76.9 mg/m^2^ for 5 days every 3 weeks)	Suspected	180	Euthanasia due to clinical signs of relapse
**English Bulldog**	F	Y	4	25.5	Obtundedmental status	Right circling, left proprioceptive deficits, decreased menace response OS	Left cerebellum	1	Anaplastic oligodendroglioma grade III	**1st** Temozolomide (160.9 mg/m^2^ for 5 days every 3 weeks)	CT confirmed	120	Euthanasia due to tumour regrowth
**French Bulldog**	M	N	5	13.3	Seizures	Mild right proprioceptive deficits and decreased menace response OD	Left frontal lobe	1	Oligodendroglioma grade II	**1st** Temozolomide 181.8 mg/m^2^ for 5 days every 3 weeks) **2nd** Lomustine (70 mg/m^2^ every 3 weeks)	CT confirmed	240	Euthanasia due to tumour regrowth
**Boxer**	F	N	9	28.5	Seizures	Lumbosacral pain	Right occipital lobe	2	**1st** Anaplastic oligodendroglioma grade III **2nd** Gliosarcoma grade IV	**1st** Temozolomide (76.9 mg/m^2^ for 5 días every 3 weeks) **2nd** Toceranib phosphate (2.8 mg/kg 3 times weekly)	CT & biopsy confirmed	241	Euthanasia due to tumour regrowth
**French Bulldog**	F	Y	8	8.9	Seizures	Decrease pupillary reflex OD	Right olfactory lobe	2	**1st** Anaplastic oligodendroglioma grade III **2nd** Anaplastic oligodendroglioma grade III	Temozolomide (93.0 mg/m^2^ for 5 days every 3 weeks)	CT & biopsy confirmed	428	Euthanasia due to aspiration pneumonia two days after the second surgery. CT ruled out IC haemorrhage
**Boxer mix**	F	Y	8	21.3	Seizures	Mild left proprioceptive deficits	Left frontal lobe	2	**1st** Oligodendroglioma grade II **2nd** Oligodendroglioma grade II	**1st** Temozolomide (76.4 mg/m^2^ for 5 days every 3 weeks) **2nd** Lomustine (70 mg/m^2^ every 3 weeks)	CT & biopsy confirmed	468	Euthanasia due to tumour regrowth
**French Bulldog**	F	Y	8	14.5	Seizures	Left pelvic limb proprioceptive deficits and decreased menace response OS	Right parietal lobe	3	**1st** Anaplastic oligodendroglioma grade III **2nd** Anaplastic oligodendroglioma grade III 3rd Anaplastic oligodendroglioma grade III	**1st** Temozolomide (81.6 mg/m^2^ for 5 days every 3 weeks) **2nd** Toceranib phosphate (2.5 mg/kg 3 times weekly) 3rd Lomustine (70 mg/m^2^ every 3 weeks)	CT & biopsy confirmed	780	Euthanasia due to tumour regrowth

kg = kilograms; n = number; F = female; M = male; Y = yes; N = no; yrs = years; OS = left eye; OD = right eye, CT = computed tomography, IC = intracranial haemorrhage.

## Data Availability

All available data is contained within the article and tables.
